# Mechanical Properties of the Pt-CNT Composite under Uniaxial Deformation: Tension and Compression

**DOI:** 10.3390/ma16114140

**Published:** 2023-06-01

**Authors:** Ustina I. Yankovaskaya, Elena A. Korznikova, Sofia D. Korpusova, Pavel V. Zakharov

**Affiliations:** 1 Department of Physics, Polzunov Altai State Technical University, Barnaul 656038, Russia; zalaevau@gmail.com; 2 Polytechnic Institute (Branch) in Mirny, North-Eastern Federal University, Mirny 678170, Russia; 3 Institute for Metals Superplasticity Problems of RAS, Ufa 450001, Russia; 4 Department of Physics, Peter the Great St. Petersburg Polytechnic University, St. Petersburg 195251, Russia; sophy-spb@yandex.ru (S.D.K.); zakharovpvl@rambler.ru (P.V.Z.)

**Keywords:** composites, modeling, molecular dynamics method, LAMMPS, reinforcement, CNT

## Abstract

Composite materials are gaining increasing attention from researchers worldwide due to their ability to offer tailored properties for various technical challenges. One of these promising fields is metal matrix composites, including carbon-reinforced metals and alloys. These materials allow for the reduction of density while simultaneously enhancing their functional properties. This study is focused on the Pt-CNT composite, its mechanical characteristics, and structural features under uniaxial deformation depending on temperature and mass fractions of carbon nanotube (CNT). The mechanical behavior of platinum reinforced with carbon nanotubes of diameters varying in the interval 6.62–16.55 Å under uniaxial tension and compression deformation has been studied by the molecular dynamics method. Simulations for tensile and compression deformations have been done for all specimens at different temperatures (viz. 300 K, 500 K, 700 K, 900 K, 1100 K, and 1500 K). The calculated mechanical characteristics allow us to conclude that, compared to pure platinum, the Young’s modulus increased by about 60%. The results indicate that yield and tensile strength values decreases with increase in temperature for all simulation blocks. This increase was due to the inherent high axial rigidity of CNTs. In this work, these characteristics are calculated for the first time for Pt-CNT. It can be concluded that CNTs can be an effective reinforcing material for composites based on a metal matrix under tensile strain.

## 1. Introduction

Metal—matrix composites are an actively developing type of material due to their promising set of properties. Carbon nanotube (CNT) is one of the most common constructive materials placed in the metal matrix used for strengthening. Among different metal-CNT combinations, one can distinguish Pt-CNT composite due to their unique set of properties and application potential. For instance, in the discussed material in fuel cells, Pt is commonly used as a catalyst for the oxygen reduction reaction (ORR) [1]. The use of Pt-CNT composites as an ORR catalyst has been shown to improve the durability and activity of the catalyst, as well as reduce the amount of Pt required [2]. Pt-CNT composites have also been explored for other catalytic reactions, such as the methanol oxidation reaction (MOR) [3]. In addition, Pt-CNT composites have the potential for sensing applications, such as the detection of hydrogen gas [4]. The high surface area and conductivity of CNTs can enhance the sensitivity and selectivity of Pt-CNT sensors. Furthermore, Pt-CNT composites have shown promise for biomedical applications, such as biosensors and drug delivery systems [5]. 

This work deals with the composite, which is a Pt metal matrix with carbon nanotubes placed in it, and its contribution to the mechanical properties of the composite under uniaxial compression/tension deformation. This approach is widely used for structural-aimed materials, especially for Al, Ni, Ti, Cu, and other ones with more complicated structures. They are interesting because of a significant change in their strength properties in comparison with monoatomic materials or other metal composites.

There are a number of fundamental problems, connected with the production of metal-CNT composites [1,2,3,4,5]. Among them, difficulties are associated with control of set mechanical properties, relatively poor CNT dispersion in the metal matrix, and their agglomerations. The low adhesion of the metal and nanotubes is also worth mentioning alone. All these issues require a vast amount of knowledge about the behavior of materials in different conditions. First of all, characteristics of metal-CNT interaction and their effect on the mechanical behavior of metal nanocomposites under strain are of crucial importance to the nanoscale.

The decision to use CNT as a reinforcing element is not a matter of choice, because they possess outstanding strength and rigidity, are resistant to high temperatures, and are open to manufacturing. All these features can increase the target properties of structural composites. These properties most notably involve high rigidity, low density, good thermal conductivity, and corrosion resistance [6].

Adding the CNT allows control of the size of metal matrix grains and increases stability under the heat treatment fixing the grain boundaries. Such results are shown by experiments for the Ni/CNT composite in the works [7,8,9]. Qualitative CNT dispersion in layered composite Cu/CNT has allowed it to achieve high strength. The layered structure has provided plasticity of the composite [10]. Thus, the importance of control of the CNT distribution in the metal matrix is highlighted.

The majority of work on this topic is focused on different aluminum composites reinforced with CNT. There is an obvious interest in such compounds as aluminum alloys are widely used in different technical devices: from household to space-related ones. As an example, the CNT role in different conditions and their contribution to the composites’ properties are investigated. The hardness of the Al/CNT composite significantly rises depending on the mass fraction of CNT in it, for instance. The authors of the work [11] have figured out the percentage of the CNT for the maximum hardness of the material—1.5%. This feature is three times higher than the hardness of pure aluminum. These results are also caused by good Al-CNT adhesion apart from other factors. The work [12] is focused on this topic. It studies not only the hardness of the alloy but also its rigidity. The authors have put pressure on the side of a voluminous cell and monitored the deformation. The results have shown that the more the density of the CNT in the aluminum, the higher rigidity of a composite and the lower the mass of the component. 

Thermal conductivity is another important feature of composites. This issue has been approached in the work [13], which is aimed at revealing the composite thermal conductivity dependence on deformation, i.e., the connection between thermal and mechanical properties. Thermal conductivity is measured before and after tensile tests to identify the dependence of deformation on thermal conductivity. Experimental results are consistent with theoretical ones. 

The destruction mechanism is a crucial stage in the process of deformation of such samples. This aspect has been studied in the work [14]. The research reveals the destruction mechanism via the formation of cracks in the metal matrix and their further enlarging to voids. 

Thus, metal/CNT composite takes the upper hand over pure metals and metal composites in their mechanical characteristics. Having a Pt for reinforcing, we have paid attention to the fact that it is used not only as functional but also as a structural material, especially in medicine. Platinum is widely used for microelectrodes in various conditions. Oxidation and, consequently, destruction frequently happen due to oxide film formation [15,16,17,18,19]. The modification of electrodes and CNT implants can improve their mechanical and functional characteristics. There are some works that describe such an approach. In the work [20,21], carbon electrodes are modified with platinum–iridium alloy. Such devices possess unique flexibility and necessary physical and technical properties [22,23]. Thus, the modification of platinum by carbon nanotubes is an actual problem. This is realized by the work [24], penetrating the issues of catalysts based on Pt/CNT, as well as by the work [25], where the use of CNTs as a reinforcing element for polymers is applicable.

Experimental methods of research are often time and effort consuming, and they do not reveal the kinetics of the processes at the atomic level. Studying many phenomena is now on a virtual plane since supercomputers and high-performing numerical methods have appeared. Atomistic modeling methods are generally accepted in addition to experiments and theory nowadays. Numerical methods are widely used as supportive tools for predicting and assessing laboratory measurements.

There are two main methods at the nanoscale: ab initio and molecular dynamics modeling (MD). Although ab initio methods can be more informative than MD in terms of interatomic interactions, they are still limited by systems with relatively small numbers of particles, measured in tens and hundreds of atoms. In contrast, MD modeling is able to operate with hundreds of thousands of particles, which is convenient for understanding mechanisms of material behavior at the nanoscale and for nanocomposite mechanical properties assessment. 

Molecular dynamics are well suited to calculate the metal-CNT interaction at the nanoscale. A large number of publications cover this topic, and they are focused on computer modeling [26,27,28,29,30]. Due to the atomic resolution, MD is also used for developing and testing new composite nanostructures with better interphase adhesion and/or nanofillers dispersion. For example, the authors of [31,32] have tested new constructions of matrixes in graphene-Al and graphene-Ni nanocomposites, using MD. In the work [33], the Ni-coverage of graphene nanofiller has been tested. Furthermore, in the work [34], the MD method has been used for the in-depth study of aluminum–graphene interaction morphology. Graphene has been used as a nanofiller for Cu-nanocomposites for testing the effect of porosity on interfacial adhesion by researchers in [35]. Moreover, Song and others [36] have carried out the MD modeling for studying the Ni-coverage effect on the mechanical behavior of single-walled CNT and composites with an embedded aluminum matrix. The result is that in the Ni-covered CNT-Al composite, Young’s modulus is notably higher than the one for pure CNT-Al composite, despite the fact that Young’s modulus for Ni-covered CNT is much lower than for CNT without coverage. They pointed out that the rising load transfer between CNT and aluminum matrix in the composite via Ni-coverage can be an efficient way. The results also show that nanotubes’ Ni-coverage sharply increases their interfacial connection to the aluminum matrix. In [37], the composite CNT-Al material’s mechanical behavior under compression is assessed using the MD-modeling method. 

The majority of the works above are devoted to the metal-CNT composite’s behavior under tension analysis. However, nanocomposites, which are used for devices and systems, can undergo either tension or compression. Actually, the behavior of CNT-metal composites for many metals under compression is studied much less than that under tension. In addition, CNT can be sensitive to warping and bending under compression. Here, we conducted a study both in nature and in the collection, which allows us to evaluate the mechanical properties of the composite under various conditions in one work, which are often used in the works described above.

Studies of Pt-CNT metal matrix composites where the CNTs have a different radius close to the experimental scale or high content (volume fraction) that are advantageous as lightweight materials have been insufficient. Thus, in this work, the Pt metal matrix composite with CNT reinforcement is studied with the use of the molecular dynamics method. The study of the composite mechanical properties under compression/tension deformation with different temperatures, for various sizes and quantities in the set of defect-free cells of reinforcing CNT is carried out. In addition, the present study will be useful in the development of Pt-CNT composites by presenting a predictive model of stiffness.

## 2. Materials and Methods

The model was based on a face-centered cubic platinum crystal with the lattice constant a = 3.920 Å. The linear dimensions were 62.1507 Å × 62.1507 Å × 62.1507 Å. The X, Y, and Z axes were placed along the crystallographic directions [100], [010], [001]. Periodic boundary conditions are imposed on all three-dimensional directions. The total number of particles ranged from 15,888 to 16,272 depending on the size of CNT and their quantity.

**Figure 1:**
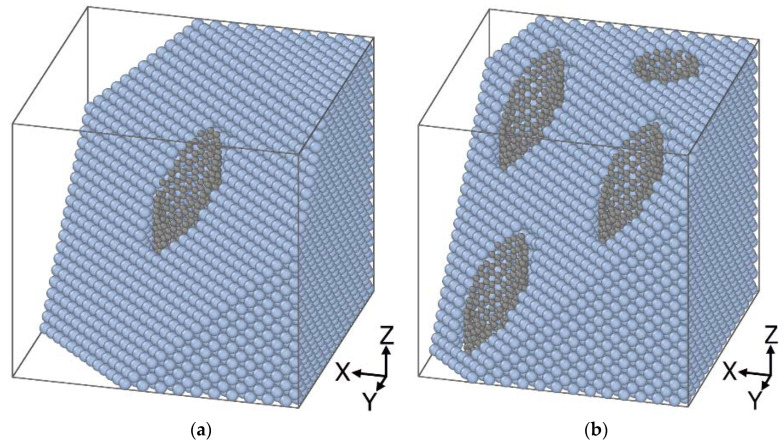
Fragment of a Pt crystal model reinforced with CNT: (**a**) the model with one CNT; (**b**) four CNT in the model block.

To study the effect of the temperature, a monocrystal metal block was integrated with the single-layered carbon nanotube of the “zigzag” type along the Z axes (Figure 1a) with the chiral indexes (0.8), length L = 62.15 Å and diameters D 6.62; 9.93; 13.24 and 16.55 Å. A cylindrical hole was “drilled” along the entire height of the Pt monocrystal, and all metal atoms inside a set cylindrical space were removed to reach such a configuration and to prevent premature Pt-Pt, C-C, and Pt-C connection breaks. Then, the hole was filled with CNT, so the 3 Å distance between C and Pt atoms along the interface of the nanocomposite was provided. These mutual configuration parameters showed a minimum of energy.

Models with different quantities of CNT were further considered (Figure 1b). A system with one, two, or four CNTs was considered. That was also done for assessment of the contribution of the mass and volume fraction to the mechanical properties of crystals. The Pt-CNT nanocomposite model with a different number of nanotubes involved CNT of the same diameter and chirality (0.16).

Table 1 shows the volume and mass fractions of carbon nanotubes for all models used in this study.

Periodic boundary conditions were applied in all directions to get the volumetric properties without Pt-CNT composite edge surface effects. The main structure constructions for modeling were done according to the Atomsk program [38]. 

LAMMPS [39] was used as a software package for calculations by the molecular dynamics method. It has the required functionality for this work and further nanocomposite deformation analysis. Previously, it has demonstrated itself as an effective tool for analyzing various aspects of structure transformation crystal lattices as a result of external influences, including the study of defects of dynamic and topological defects [40,41,42], resistance to heating nanocrystals reinforced with carbon nanotubes [43], analysis of deformation-induced phase transitions [44], shape changes crystals as a result of external electromagnetic influences [45], and many others. The MEAM potential was chosen to describe the Pt-Pt and Pt-CNT interaction. The Pt-CNT system interatomic potential of the considered composite was developed on the basis of the formalism of the second modified method of the nested nearest neighbor atom (2NN MEAM) [46].

**Figure 2:**
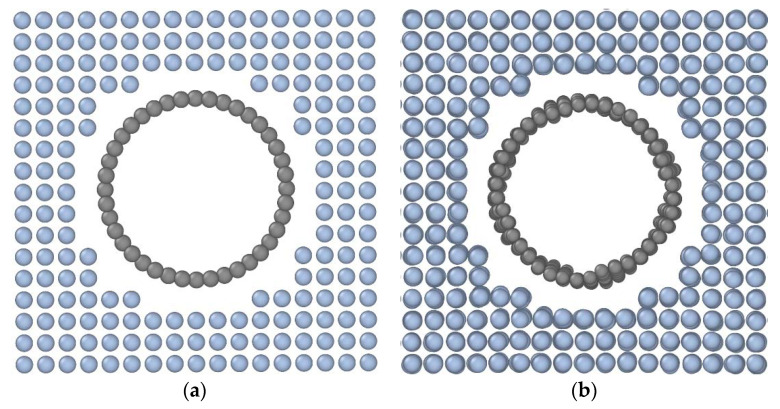
The model of a Pt-CNT system before (**a**) and after (**b**) structure relaxation.

At the initial stage, the model energy minimization was made with further relaxation in 1 ns with a set temperature and zero pressure. It helped to get rid of extra stress and to reach a more stable crystal structure state. OVITO [47] was used for the calculation results from visualization and their graphical representation for subsequent analysis. In Figure 2, there is an example of a simulation model of a Pt-CNT composite after the primary relaxation.

The stress along the z ([001]) direction of the deformation speed 10^−9^/ps was introduced to the composite during the deforming load modeling. This speed is lower than the typical one for other similar models [48,49,50]. It was done to balance the calculation time, taking into account the problems of the amorphous disorder unintended deformation with a high speed and spending on the calculation with a low speed [48]. 

When modeling Pt-CNT composite uniaxial tension, the NVT ensemble was used. It is normally applied at a 300 K temperature. The time step was reduced to 0.5 fs in order to avoid errors in calculating of CNT atom’s trajectory during the destruction. 

Mechanical stresses are calculated on the basis of virial stress in the same way as in [48]:

(1) $$\sigma \left(r\right)=\frac{1}{\Omega }\sum_{i}\left[-m_{i}{\dot{u}}_{i}⨂{\dot{u}}_{i}+\frac{1}{2}\sum_{i\neq j}r_{ij}⨂f_{ij}\right]$$

*Ω*—the total volume; *m_i_*—the *i* atom mass; *u_i_*—the time derivative, which refers to the displacement vector of the *i* atom relative to the initial position; *r_ij_*—the distance between the position vectors *r_i_* and *r_j_* of atoms *i* and *j*, respectively; *f_ij_*—interatomic force acting on atom *i* from atom *j* [22]. Ω is calculated in a balanced state, and the Pt-CNT composite total volume is calculated as a sum of platinum with a hole and CNT volumes. The compression/tension modeling has been performed until the ε deformation reaches the limit values of 0.25/0.45. The calculations were carried out in the context of the canonical NVT ensemble with the temperature range 300–1700 K, with a time step of 0.5 fs.

## 3. Results and Discussion

The number of CNT, their volume fraction, and their diameter in the metal matrix composites have a great influence on the nanocomposites’ mechanical properties. The MD-modeling method studies show that CNT has an obvious strengthening effect on the metal matrix composites [2,3,4]. It is considered that a good reinforcing effect of the metal matrix composite carbon nanotubes is connected with the high bearing capacity of CNT due to their high strength and surface area [5,6,7].

### 3.1. The Role of the CNT Diameter

The diameter of CNT is one of the influences on the mechanical properties of composite materials.

**Figure 3:**
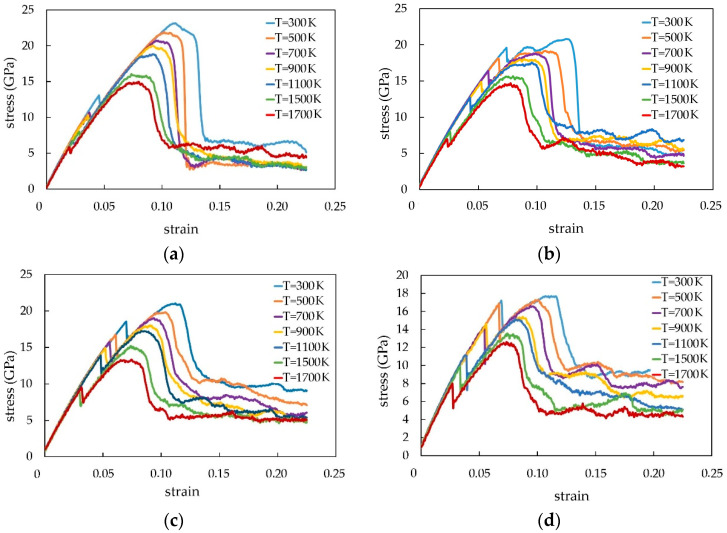

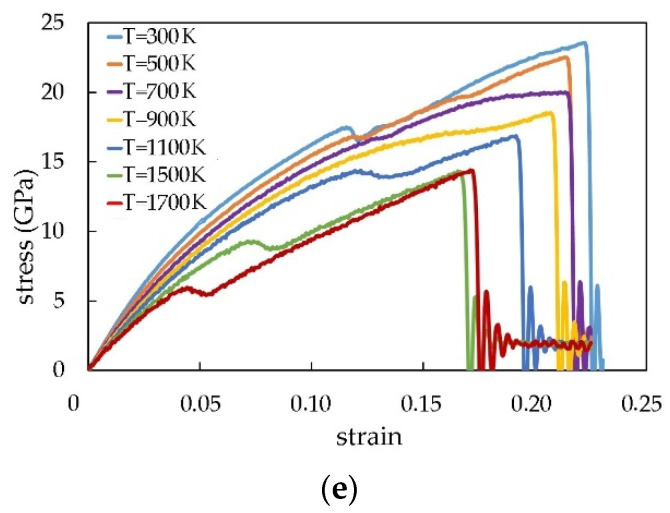
Stress/strain dependence under the uniaxial tensile load of the Pt-CNT nanocomposite at different temperatures: (**a**)—d = 6.62 Å; (**b**)—d = 9.93 Å; (**c**)—d = 13.24 Å; (**d**)—d = 16.55 Å, (**e**)—pure Pt.

In Figure 3, there is a stress–strain tension dependence of the Pt-CNT composite for models with different CNT diameters under various temperatures and also for pure platinum.

All these curves have a linear section at the beginning representing an elastic area. After reaching the maximum value, the stress decreases sharply, which indicates the material entry into the plastic area. It has been noted that the inclines of the stress–strain curves for models with different CNT diameters decrease with the temperature rising. It is caused by the thermal fluctuations that increase the speeds of atoms under high temperatures and make the nanocomposites much softer [6]. To understand the effect of the temperature and carbon nanotube diameter on the Pt-CNT composite mechanical properties clearly, the values of Young’s modulus, strength limit, destruction deformation, and yield stress are measured for the studied composite model under the tensile stress. They are shown in Table 2.

**Figure 4:**
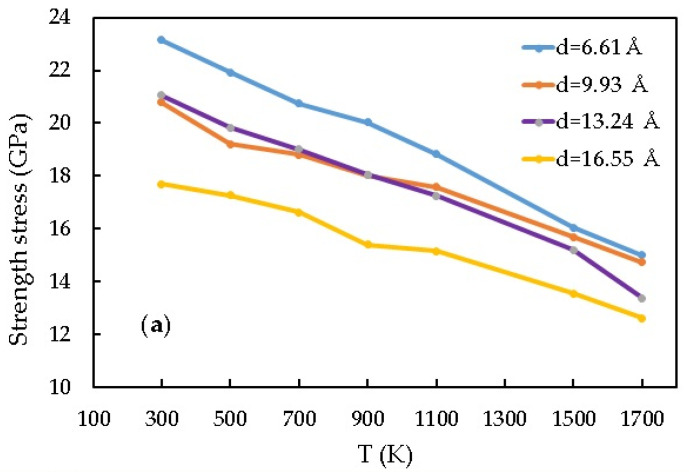

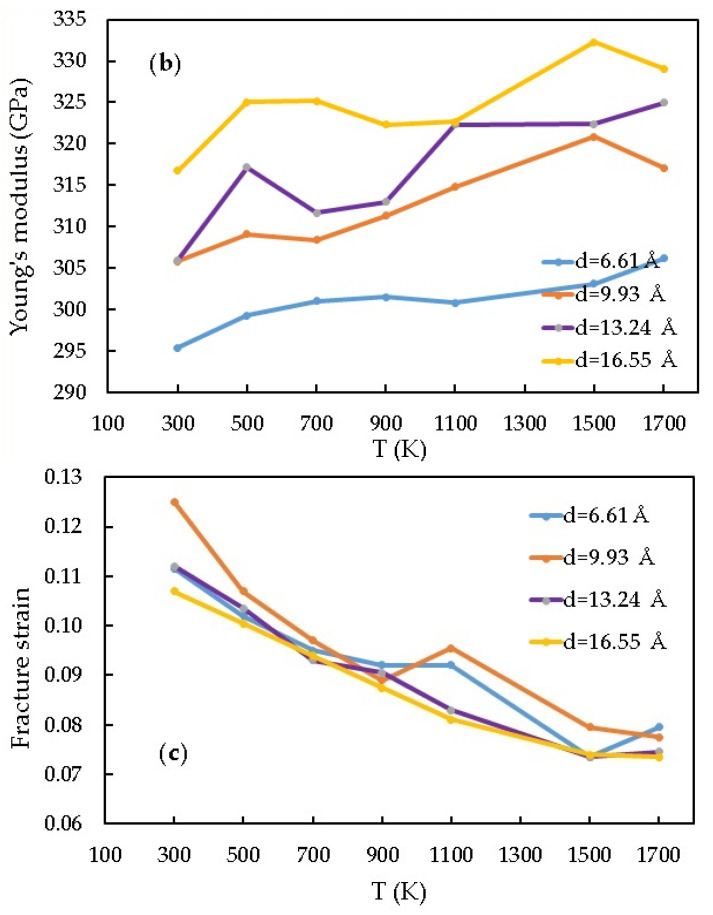
The Pt-CNT composite mechanical properties at different temperatures under uniaxial tension: (**a**) strength stress, (**b**) Young’s modulus, and (**c**) destruction deformation.

The results (Table 2) of measuring Young’s modulus, strength limit, destruction deformation, and yield stress of the Pt-CNT composite at different temperatures are shown in Figure 4.

The authors of the work [7] have found that there is an optimal CNT diameter for the composite. If the diameter of CNT is too small, there is no great effect of the reinforcement. If the diameter is too large, the stability of the composite system decreases, and the composite becomes unstable under deformation. 

The highest value of Young’s modulus under tensile load, equal to 332.24 GPa, is measured with the CNT diameter = 16.55 Å (with the mass fraction 7.05%) and temperature 1500 K. The growth of the elastic modulus values as a function of a temperature for all diameters is 3.5–3.7%. The larger the diameter, the higher the modulus. Note that the maximum values of Young’s modulus are marked at a temperature of 1500 K for almost all models.

As we can see in Figure 4c, the tensile strength limit of the Pt-CNT nanocomposite with a smaller nanotube diameter is 23.6% higher than the one with a larger diameter. It is caused by changes in both bond length and bond angle along the tension direction of CNT [8].

Figure 4a shows that the values of destruction deformation are comparable for Pt-CNT nanocomposite models with different diameters. Only the model with a CNT diameter = 9.93 Å (volume fraction is 4.13%) has a higher value at all temperatures studied and for the model with a CNT diameter = 6.62 Å (volume fraction is 2.76%), there is an increase in destruction deformation values in the temperature range from 1100 K.

Under the compression, the results are as follows: Young’s modulus value maximum increase for the composite in comparison with the monocrystal is observed with the CNT diameter = 6.62 Å (volume fraction is of 2.76%), (Young’s modulus increases by more than 60%) (Table 3). 

**Figure 5:**
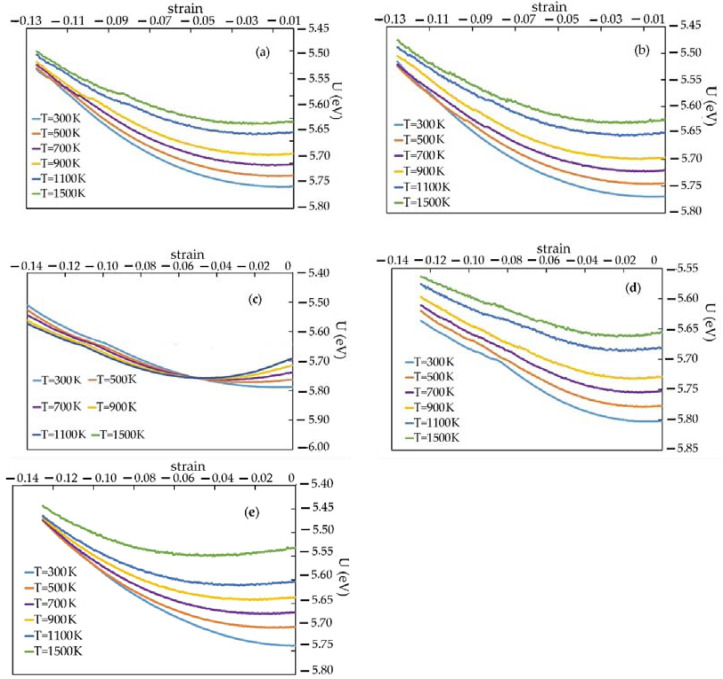
The Pt-CNT composite energy at different temperatures under the uniaxial compression: (**a**) d = 6.62 Å (v.f. 2.76%), (**b**) d = 9.93 Å (4.13%), (**c**) d = 13.24 Å (5.54%), (**d**) d = 16.55 Å (7.05 %), and (**e**) pure Pt.

Figure 5 shows the change of the total energy U with the applied compressive strain for the CNT diameter equal to 6.62 Å (volume fraction is of 2.76%), 9.93 Å (4.13%), 13.24 Å (5.54%), 16.55 Å (7.05%), and pure Pt.

The trend on all charts shows a general nature. We note that for detection with d = 13.24 Å, the structure turned out to be less stable at a temperature close to 1500. The most resistance to deformation is the pronounced signs associated with the transformation of the structure with less deformation of the skin.

Under compression deformation, the total energy of the nanocomposite changes mainly quadratically. Besides, the energy changes the shape of the curve when certain deformation values are reached. In the range of deformations between these differences, the main trajectories (quadratic U(ε)) show the phase of elastic deformation (linear change σ(ε)), and the differences U(ε) show the beginning of plastic deformation (elasticity) of the metal matrix.

**Figure 6:**
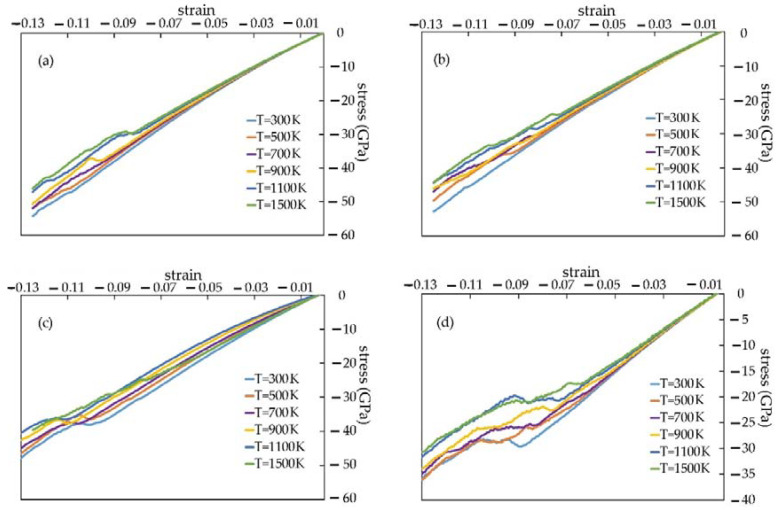
The stress/strain curves of the Pt-CNT nanocomposite under the uniaxial compression at different temperatures and CNT diameters: (**a**) d = 6.62 Å, (**b**) d = 9.93 Å, (**c**) d = 13.24 Å, and (**d**) d = 16.55 Å.

In Figure 6, there are the stress/strain curves of the Pt-CNT nanocomposite under the uniaxial compression at different temperatures and CNT diameters. It can be noted that there is a 60% increase in Young’s modulus in comparison to the “pure platinum” crystal. Thus, the use of CNT leads to a significant rise in composite rigidity.

### 3.2. The Role of the CNT Number in the Model

The mechanical properties of the CNT/metal composites can be improved by increasing the number of nanotubes. There are models of the composite with different numbers of nanotubes (one and four) in Figure 1. The diameter of carbon nanotubes is the same: 13.24 Å.

**Figure 7:**
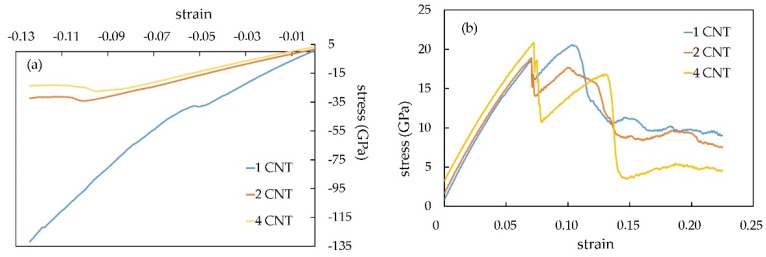
The stress/strain dependences of the Pt-CNT composite for the model with a different number of CNT at T = 300 K: (**a**) compression; (**b**) tension.

**Figure 8:**
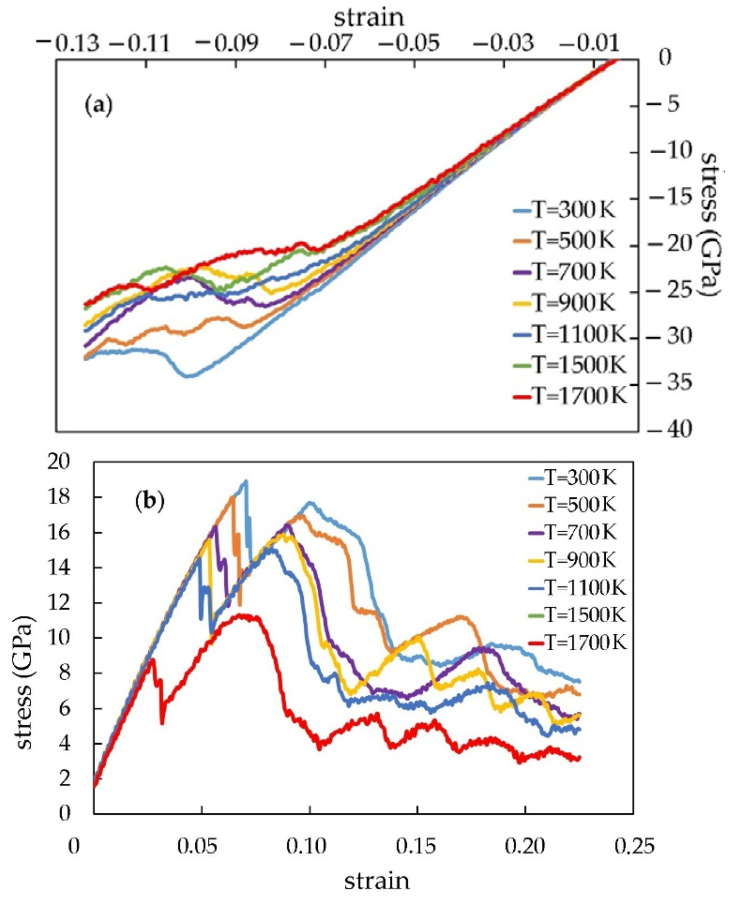
The stress/strain dependences of the Pt-CNT composite for the model with 2 CNT at different temperatures: (**a**) compression; (**b**) tension.

Figure 7 shows the stress/strain dependences of the composite, reinforced with 1, 2, and 4 CNTs at 300 K temperature. Figure 8 demonstrates the stress/strain dependence at different temperatures with an example of a composite with two CNTs.

During modeling Young’s modulus and the yield stress under compressive and tensile loads were calculated, and the results are shown in Table 4.

If we compare Pt-CNT nanocomposite stress-strain curves with different CNT numbers, we will see that the maximum tensile and compressive stress is much higher for the model with four nanotubes. For example, the maximum tensile stress value for a model with 4 nanotubes is 20.83 GPa at a T = 300 K, while the same for a model with 2 nanotubes is 20.79 GPa. The difference proves that the CNT number affects the mechanical properties of the composite. 

**Figure 9:**
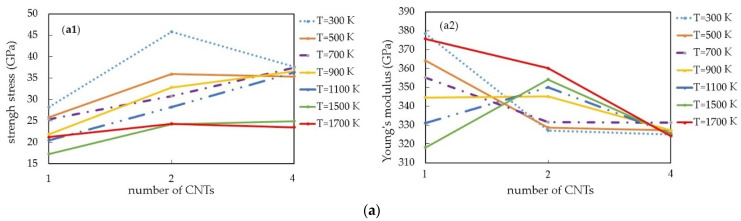

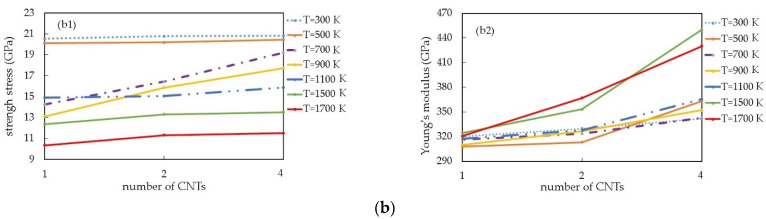
The Pt-CNT composite mechanical properties with different CNT numbers and temperatures: (**a**) compression; (**b**) tension.

Figure 9 demonstrates the mechanical properties of the Pt-CNT nanocomposite depending on the number of CNTs at different temperatures.

According to the calculations, Young’s modulus under the tensile stress for 2 CNT is 11.52% higher than for 1 CNT. The elastic modulus has improved by 27.9% for the composite with 4 CNT. As for the strength limit, it improves with the CNT number increase at a temperature range of 700–1100 K (it is 25.9% higher for the model with 4 CNT than for one with 1 CNT). The situation is quite different for the compression. The maximum value of Young’s modulus is higher for the model with 1 CNT, but the maximum value of the tensile strength is observed for the model with 2 CNT.

Figure 9 shows the influence of the CNT mass fraction on the CNT/metal composites’ mechanical properties. It is clear that in the Pt-CNT composite, Young’s modulus and strength limit increase together with the CNT volume fraction (23.05%). There is a linear dependence between the mechanical properties and the CNT number during tension. The larger the number of CNT, the higher the strength limit of the CNT/metal composites (an increase from 14.92 to 20.91 GPa). Young’s modulus and the strength limit of a composite increase together with the CNT volume fraction, as has already been mentioned, from 320.78 GPa (for the model with 1 CNT) to 449.99 GPa (for the model with 4 CNT). Consequently, the CNT volume fraction increase will improve the mechanical properties of composites. The maximum value of Young’s modulus under the compressive strain is observed for the model with 1 CNT at a temperature range of 300–700 K. The situation changes further. The 2 CNT model has a higher strength limit and Young’s modulus value at higher temperatures.

### 3.3. Destruction Mechanism 

**Figure 10:**
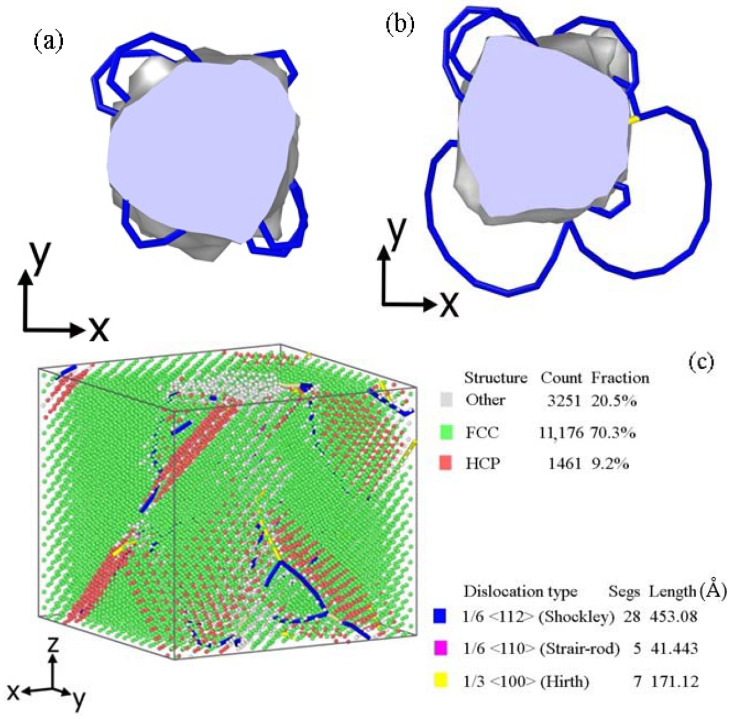
The stages of the cell (CNT 20, 700 K) destruction via the growth of dislocation loops under the compression deformation. ɛ = 0.16 (**a**), ɛ = 0.17 (**b**), ɛ = 0.2 (**c**).

The destruction mechanism of a nanotube under compression deformation is similar to the annihilation mechanism of cavities under intensive external influence [51]. There is a dislocation structure development process for deformation ɛ = 0.16 (a) and ɛ = 0.17 (b) in Figure 10. The nanotube with a diameter of 13.24 Å is used as an example. Partial Shockley’s dislocations in the form of dislocation loops begin to form actively. They interact with each other further due to periodic boundary conditions. Total destruction of a nanotube, followed by the phase composition change along the dislocations and movement of atoms, occurs under the compression deformation ɛ = 0.2 (Figure 10c).

**Figure 11:**
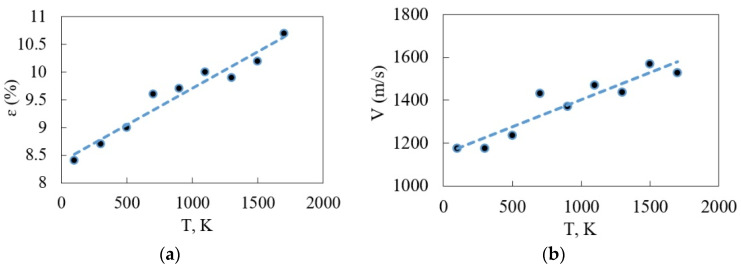
Behavior of dislocations depending on temperature: (**a**) the dependence of the relative elongation values with the first Shockley’s dislocations on temperature; (**b**) the dependence of the dislocation loop speed on temperature.

Due to the assessment of the temperature role in this process, it is found that first dislocations appear later with an increase in the temperature. Figure 11 shows the deformation-temperature dependence. Note that the first appearance of dislocations was carried out at a relative deformation ɛ = 8.4%. Subsequently, a linear increase of up to 11% was observed. This is due to the increase in elasticity with increasing temperature for the considered composite. The data can be correlated with Figure 4a, which shows characteristic curves for the reduction in modulus of elasticity.

Dislocation behavior has been looked at closer. The dependence of the dislocation loop speed on temperature is found. There are average values for different loops in crystals from the formation moment till the movement to the calculation cell boundary in Figure 11. A trend towards a speed rising from 1200 m/s to 1600 m/s in the set temperature range is common. In general, the tendency is natural and is observed in acceptable speed intervals. It is a well-known fact that the speed of dislocations cannot exceed the speed of sound in a crystal. According to our estimates, it is about 2800 m/s for this model. The dislocation length rising speed for the considered crystal is also assessed. It is almost the same for all set temperature ranges. It means that the dislocation net turns to be more brunched at lower temperatures because the dislocation loops move slower. The average dislocation length rising speed is 4300 m/s.

**Figure 12:**
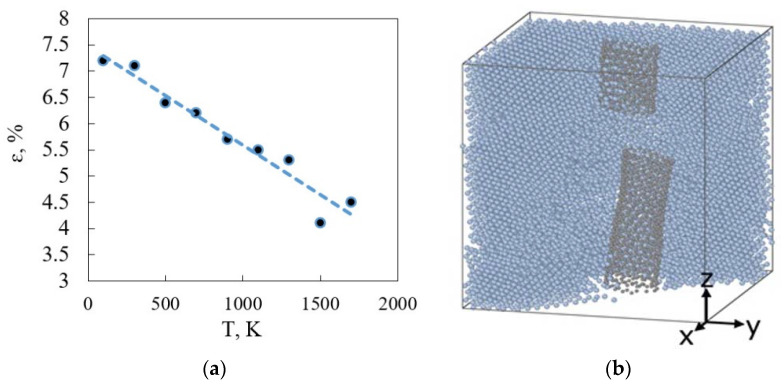
The CNT destruction under tension: (**a**) the dependence of the relative elongation on the temperature, which causes CNT break; (**b**) CNT break visualization.

We then focus on the role of temperature under tension deformation. There is a steady decrease in relative deformation value with temperature rise (Figure 12a). The CNT breaks with this deformation value.

**Figure 13:**
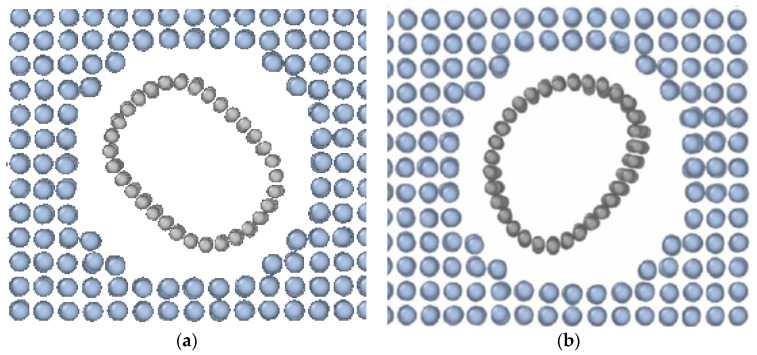
The CNT deformation: (**a**) symmetrical; (**b**) asymmetrical.

The CNT cross-section shape changes because of deformation. The symmetrical separation from the metal (Figure 13a) is possible at low temperatures (up to 300 K). The separation of one CNT side from the platinum matrix (Figure 13b) is mainly observed with temperature rise.

## 4. Conclusions

In summary, this study used the molecular dynamics method to investigate the mechanical properties of platinum reinforced with carbon nanotubes under uniaxial compression/tension deformation. The results demonstrate that the addition of carbon nanotubes significantly enhances Young’s modulus of the composite material by approximately 60%, mainly due to the axial rigidity of the nanotubes. However, the yield stress and yield strain of the Pt-CNT composite do not increase compared to pure platinum, while the strength and yield strain decrease under compression load due to the rise of free volume in the material caused by the presence of nanotubes. The density of nanotubes also plays a crucial role, as a higher CNT density results in less efficient mechanical parameters.

Interestingly, the study shows that the increase of CNT diameter from 6.62 to 16.55 Å results in a moderate growth of both compressional and tensional stress to about 20%. Moreover, the temperature during tension has a significant influence on the relative elongation and can cause a CNT break, leading to a decrease in relative elongation by almost half.

Overall, this study’s findings provide valuable insights into the mechanical properties of composite materials reinforced with carbon nanotubes, which can guide the development of new high-performance materials for various applications. The study’s significance lies in its potential to optimize the material’s properties by selecting the appropriate diameter and density of carbon nanotubes and controlling the temperature during tension, leading to the creation of new composite materials with improved mechanical properties.

## Figures and Tables

**Table 1:** Volume and mass fractions of CNT for different models.

	Model	Volume Fraction of CNT (%)	Mass Fraction of CNT (%)
Different diameters of CNT (Å)	6.62	2.76	0.17
	9.93	4.13	0.26
	13.24	5.54	0.36
	16.55	7.05	0.46
Different quantities of CNT	1	5.54	0.36
	2	11.2	0.77
	4	23.05	1.81

**Table 2:** The mechanical properties of the Pt-CNT composite under the uniaxial tension (with various temperatures and CNT diameters).

CNT Diameter (Å)	T (K)	Young’s Modulus (GPa)	Strength Stress (GPa)	Destruction Deformation (%)	Yield Stress (GPa)
6.62	300	295.42	23.15	0.11	23.11
	500	299.28	21.93	0.10	21.54
	700	301.04	20.73	0.10	20.60
	900	301.49	20.03	0.09	18.21
	1100	300.79	18.83	0.09	16.34
	1500	303.10	16.05	0.07	15.64
	1700	306.16	14.98	0.08	11.45
9.93	300	305.83	20.79	0.125	19.61
	500	309.08	19.20	0.107	18.76
	700	308.43	18.80	0.097	17.31
	900	311.29	18.03	0.089	17.93
	1100	314.81	17.58	0.0955	17.23
	1500	320.83	15.68	0.0795	15.52
	1700	317.12	14.72	0.0775	14.25
13.24	300	305.98	21.06	0.11	20.50
	500	317.18	19.82	0.10	19.64
	700	311.70	19.01	0.09	18.03
	900	313.01	18.05	0.09	17.16
	1100	322.28	17.25	0.08	16.89
	1500	322.40	15.19	0.07	12.76
	1700	324.96	13.36	0.07	9.87
16.55	300	316.76	17.69	0.107	14.32
	500	325.01	17.27	0.1005	15.83
	700	325.17	16.62	0.094	13.7
	900	322.31	15.39	0.0875	13.84
	1100	322.70	15.15	0.081	13.69
	1500	332.24	13.54	0.074	8.27
	1700	329.04	12.61	0.0735	12.32
Pure Pt	300	179.32	23.56	0.22	17.36
	500	167.52	22.53	0.213	16.73
	700	158.86	20.04	0.2135	16.7
	900	156.44	18.54	0.207	17.11
	1100	152.52	16.88	0.1915	14.37
	1500	153.31	14.36	0.1655	9.3
	1700	154.07	14.44	0.171	5.8

**Table 3:** The calculation results of the Pt-CNT composite mechanical properties under the uniaxial compression.

CNT Diameter (Å)	T (K)	Young’s Modulus (GPa)	Strength Stress (GPa)	Destruction Deformation (%)
6.63	300	305.12	47.23	0.11
	500	308.14	46.10	0.10
	700	312.56	41.60	0.10
	900	314.21	37.16	0.10
	1100	309.57	29.67	0.09
	1500	313.25	29.28	0.07
9.93	300	327.12	45.85	0.11
	500	328.63	35.94	0.09
	700	331.52	30.79	0.08
	900	345.12	32.81	0.09
	1100	350.12	28.29	0.08
	1500	354.23	24.27	0.07
13.24	300	325.08	37.66	0.10
	500	327.18	35.38	0.10
	700	331.40	37.49	0.11
	900	327.18	36.60	0.11
	1100	325.52	36.30	0.11
	1500	325.19	24.95	0.08
16.55	300	378.62	28.26	0.09
	500	364.13	25.88	0.08
	700	355.11	25.45	0.08
	900	344.55	21.92	0.07
	1100	331.02	20.48	0.07
	1500	318.14	17.31	0.06

**Table 4:** The mechanical properties of the Pt-CNT composite with the different numbers of CNTs at various temperatures.

CNT Number	T (K)	Tension		Compression	
		Young’s Modulus (GPa)	Strength Stress (GPa)	Young’s Modulus (GPa)	Strength Stress (GPa)
1	300	320.56	20.54	375.65	21.26
	500	307.84	20.11	364.13	25.88
	700	316.32	14.24	355.11	25.45
	900	310.01	13.12	344.55	21.92
	1100	317.56	14.92	331.02	20.48
	1500	324.60	12.36	318.14	17.31
	1700	320.78	10.36	378.62	28.26
2	300	329.65	20.79	327.12	45.85
	500	313.26	20.20	328.63	35.94
	700	323.43	16.43	331.52	30.79
	900	326.65	15.87	345.12	32.81
	1100	327.94	15.07	350.12	28.29
	1500	353.32	13.32	354.23	24.27
	1700	366.89	11.32	360.20	24.34
4	300	342.02	20.83	325.08	37.66
	500	363.19	20.47	327.18	35.38
	700	342.80	19.21	331.40	37.49
	900	352.14	17.73	327.18	36.60
	1100	365.36	15.88	325.52	36.30
	1500	449.99	13.51	325.19	24.95
	1700	430.09	11.51	324.25	23.52

## Data Availability

Data is available by contacting with the corresponding author upon the reasonable request.
